# Use of Poly(vinyl alcohol) in Spray-Dried Dispersions: Enhancing Solubility and Stability of Proteolysis Targeting Chimeras

**DOI:** 10.3390/pharmaceutics16070924

**Published:** 2024-07-11

**Authors:** Lena Mareczek, Lena K. Mueller, Laura Halstenberg, Thomas M. Geiger, Michael Walz, Min Zheng, Felix Hausch

**Affiliations:** 1 Merck Life Science KGaA, 64293 Darmstadt, Germany; 2 Department of Chemistry, Technical University of Darmstadt, 64287 Darmstadt, Germany; 3 Centre for Synthetic Biology, Technical University of Darmstadt, 64287 Darmstadt, Germany

**Keywords:** PROTACs, spray drying, Poly(vinyl alcohol), PVA, protein degraders, amorphous solid dispersion, ASD, solubility enhancement

## Abstract

PROTACs, proteolysis targeting chimeras, are bifunctional molecules inducing protein degradation through a unique proximity-based mode of action. While offering several advantages unachievable by classical drugs, PROTACs have unfavorable physicochemical properties that pose challenges in application and formulation. In this study, we show the solubility enhancement of two PROTACs, ARV-110 and SelDeg51, using Poly(vinyl alcohol). Hereby, we apply a three-fluid nozzle spray drying set-up to generate an amorphous solid dispersion with a 30% *w*/*w* drug loading with the respective PROTACs and the hydrophilic polymer. Dissolution enhancement was achieved and demonstrated for t = 0 and t = 4 weeks at 5 °C using a phosphate buffer with a pH of 6.8. A pH shift study on ARV-110-PVA is shown, covering transfer from simulated gastric fluid (SGF) at pH 2.0 to fasted-state simulated intestinal fluid (FaSSIF) at pH 6.5. Additionally, activity studies and binding assays of the pure SelDeg51 versus the spray-dried SelDeg51-PVA indicate no difference between both samples. Our results show how modern enabling formulation technologies can partially alleviate challenging physicochemical properties, such as the poor solubility of increasingly large ‘small’ molecules.

## 1. Introduction

Traditionally, small-molecule drug discovery focused on structures that were able to occupy a functionally relevant and selective site of the target molecule. This limits novel drug discovery as only 25% of the human proteome is pharmaceutically accessible through tractable small-molecule binding sites. Ultimately, this would classify many disease-relevant proteins, such as transcription factors, scaffolding proteins or non-enzymatic proteins, as undruggable [1].

In the last decade, event-driven modes of action, such as proteolysis targeting chimeras (PROTACs), have emerged. PROTACs do not require functionally relevant binding sites, such as binding pockets, but are designed to target specific structures on the Protein of Interest (POI) and toward the structure involved for further degradation. They chemically induce the degradation of target proteins inside cells and, in turn, lead to the permanent inactivation of the entire protein [2].

These bifunctional molecules exhibit one ligand for the respective POI and another ligand that recruits an E3 ubiquitin ligase connected by a linker. A ternary complex between the POI and E3 ligase is formed. The POI is ubiquitinated, recognized and degraded by the 26S proteasome from the Ubiquitin Proteasome System (UPS) [3,4]. While the POI ligand is selective and unique for the specific target protein, two major compound classes for E3 ligases have emerged. While there are more than 600 E3 ligases known, PROTACs mainly address cereblon (CRBN, with Thalidomide as the lead structure) [5] or von Hippel–Lindau (VHL) as an E3 ligase [6]. There have also been reports on addressing the mouse double minute 2 homolog (MDM2) and the cellular inhibitor of apoptosis protein 1 (clAP1) [7].

However, the developability of PROTACs is challenging as these structures are well beyond the rule of five (bRo5), displaying a lower chance of desirable oral bioavailability. Lipinski‘s rule of five states that poor absorption or permeation behavior is more prevalent in molecules that exhibit more than five hydrogen bond donors (HBD > 5) and have a molecular weight greater than 500 Da (MW > 500) and a calculated logP greater than five (cLogP > 5) [8,9]. Yang et al. describe a typical PROTAC with a molecular weight > 800 Da and a predicted human dose of 200 mg [10]. In addition to being bRo5, these compounds are mostly classified in the Developability Classification System (DCS) as Class IIb or IV [10], which defines them as having poor solubility and good permeability (Class IIb) or poor solubility paired with poor permeability (Class IV). This classification system was developed as a derivative of the Biopharmaceutical Classification System (BCS) by Butler and Dressman to aid developability by incorporating biorelevant solubility while categorizing compounds regarding their solubility and permeability [11].

Due to their low solubility and non-specific binding, many of the well-established assays for the in vitro characterization of small molecules fail when applied in the evaluation and characterization of PROTACs. This also leads to challenges in permeability assays and in silico predictions that help find optimal clinical doses. Despite these undesirable properties, ARV-471 entered Phase III in clinical trials formulated as an oral solid dosage form [10], showing how high potency, as well as a prolonged pharmacodynamic effect, which might be able to counterbalance its poor solubility and permeability [9]. Additionally, several PROTACs targeting prostate cancer have entered clinical studies, including ARV-110, a further orally available PROTAC [12].

A very promising technology in the formulation of poorly soluble compounds and an opportunity for bRo5-classified PROTACs is the design of amorphous solid dispersion (ASD). ASD is composed of one or more Active Pharmaceutical Ingredients (APIs) in their amorphous forms, as well as one or more auxiliary materials, mostly polymers, that stabilize this high-energy state in a polymeric matrix. To obtain an amorphous state, the crystalline lattice needs to be disrupted either through melting or dissolving of the API. Spray drying is often used as a solvent-based technology to generate ASDs [13]. For the respective stabilizing matrix, formulators can choose from a vast variety of polymers that are mostly co-soluble with the API in a single organic solvent [14]. This set-up requires a two-fluid nozzle, where one channel forwards the organic feed containing the API and polymer, and the other forwards the spraying gas. In our previous work, we investigated the applicability of hydrophilic polymers, which require the use of a three-fluid nozzle as the API and polymer demand an individual solvent. An additional channel is present in the nozzle, which can forward the hydrophilic polymer dissolved in an aqueous liquid [15].

In this work, we follow up on our previous findings where we investigated PVA with a 30% *w*/*w* drug loading with small-molecule drugs from the BSC Classes II and IV to evaluate if our described protocol can be utilized for large and challenging structures, such as PROTACs [16]. Additionally, we follow up on experiments undertaken by Hofmann et al. In their research, they show the applicability of spray drying PROTACs and polymers to generate ASD containing the PROTAC MS4075 with Soluplus and E PO [17]. As a first step, they performed a polymer screening using a solvent-based 96 well plate set-up, which included the use of two PVA grades, PVA 4-88 and PVA 3-82. Both achieved high dissolution values in the screening but were not further investigated in spray drying, which might be due to the lack of three-fluid nozzle equipment. Here, we show the solubility enhancement of two PROTACs, the crystalline Arvinas 110 (ARV-110) and amorphous SelDeg51, using PVA 3-82 in a three-fluid nozzle spray drying set-up.

## 2. Materials and Methods

### 2.1. Materials

Poly(vinyl alcohol) 3-82 (Parteck^®^ MXP 3-82, EMPROVE^®^ ESSENTIAL, Merck KGaA, Darmstadt, Germany), Bavdegalutamide (ARV-110) (MedChemExpress, Monmouth Junction, NJ, USA), SelDeg51 (TU Darmstadt, Darmstadt, Germany), acetonitrile (Sigma Aldrich, Taufkirchen, Germany), dimethylsulfoxide (Sigma Aldrich, Taufkirchen, Germany), ethanol (Sigma Aldrich, Taufkirchen, Germany), formic acid (Sigma Aldrich, Taufkirchen, Germnay), methanol (Sigma Aldrich, Taufkirchen, Germany), phosphate buffer (pH 6.8) (protocol by USP), fasted-state simulated intestinal fluid and simulated gastric fluid (Biorelevant, London, UK) and Milli-Q^®^ Water (Merck Millipore, Burlington, MA, USA).

### 2.2. Methods

#### 2.2.1. Synthesis of SelDeg51

SelDeg51 was prepared according to the literature [18].

The corresponding azide (1.0 eq.) and alkyne [19,20] precursor (1.0 eq.) were dissolved in *tert*-butanol, water and DMSO (1:1:10). The solution was degassed using argon. Copper(II) sulfate pentahydrate (1 M in water, 0.4 eq.) and (+)-sodium L-ascorbate (1 M in water, 0.4 eq.) were added. The solution was stirred for 18 h at room temperature. DCM was added, and the mixture was washed with brine. The organic phase was dried over MgSO_4_ and concentrated under reduced pressure. The obtained product was purified by preparative reverse phase HPLC (5–100%) and dried by lyophilization.

HRMS (ESI) m/z: [M+H]^+^ calculated for C_72_H_91_FN_8_O_14_S = 1343.64323; found = 1343.64340.

LC-MS: [30–100% Solvent B, 3.0 min]: Rt = 2.2 min. >99% purity.

#### 2.2.2. Spray Drying

Equipment: SD was performed on a Buchi B295 equipped with an inert loop and high-performance cyclone (Buchi Labortechnik AG, Flawil, Switzerland).

Parameters: Inlet temperature, 90 °C and outlet temperature, 50 °C (SelDeg51). Inlet temperature, 80 °C and outlet temperature, 45 °C (ARV-110), with N_2_ as inert drying gas, flow rate of 35 m^3^/h and Aspirator at 100% and atomizing air flow rate of 670 L/min (N2 55 mm). Nozzles used: three-fluid nozzle.

Solid dispersions of ARV-110 and SelDeg51: PVA was dissolved in water, ARV-110 was dissolved in a mixture of dichloromethane and methanol in a proportion of 2:3, and SelDeg51 was dissolved in methanol. The solutions were spray dried using a three-fluid nozzle. The inner feed forwarded the API solution, and the outer feed forwarded the respective polymer solution. A syringe pump (PHD ULTRA^TM^ Syringe Pump, Harvard Apparatus, Holliston, MA, USA) was used to forward both solutions at 4 mL/min.

#### 2.2.3. Differential Scanning Calorimetry (DSC)

DSC was performed using a Mettler Toledo DSC821e (Mettler Toledo, Gießen, Germany) in N_2_ atmosphere (50.0 mL). Temperature scale was set from 30 °C to 320 °C, with a heating rate of 10 K/min.

#### 2.2.4. Scanning Electron Microscopy (SEM)

Scanning electron microscope (SEM) was performed using a Zeiss (Jena, Germany) Gemini 460: Field Emission Gun (FEG cathode), variable pressure low vacuum system, magnification 8×–2000k×, accelerating voltage 0.02 kV–30 kV, detectors: In-lens detectors, SE detector.

#### 2.2.5. X-ray Powder Diffraction (XRPD)

X-ray powder diffraction was measured using a Miniflex 600 X-ray diffractometer (Rigaku, Japan), with CuKα radiation (λ = 1.54 A), reflector mode of 3°–50° 2θ (deg), scan speed of 10° 2θ (deg) min and step size of 0.02θ (deg). Acceleration voltage is 45 kV, and 15 mA is the current.

#### 2.2.6. Dissolution

Non-sink dissolution of SelDeg51/ARV-110: The sample (5 mg of raw material or 16.67 mg of SDD (30% drug loading)) was placed in an Erlenmeyer flask. Next, 25 mL of dissolution media (phosphate buffer, pH 6.8) was added to the flask and stirred at 100 rpm on a magnetic stirrer plate. After predetermined time points (5, 10, 20, 40, 60, 90 and 120 min), a sample of 500 µL was taken from the dissolution vessel and immediately replaced with fresh dissolution media. The samples were filtered through a 0.45 µm PTFE filter and diluted with Eluent B (acetonitrile + 0.1% formic acid) (1:1). Finally, the samples were analyzed using RP-HPLC.

#### 2.2.7. Reversed-Phase High-Performance Liquid Chromatography (RP-HPLC)

Equipment: For RP-HPLC, an Agilent 1260 Infinity HPLC system (Agilent, Santa Clara, CA, USA) was equipped with an Agilent 1260 II variable wavelength detector.

RP-HPLC of SelDeg51: Column: Infinity Lab Poroshell 120 EC-C18 (2.1 × 50 mm, 2.7 µm), oven: 40 °C; mobile phase: Eluent A = 0.1% formic acid in deionized water, Eluent B = 0.1% formic acid in acetonitrile, linear gradient at 50:50 ratio Eluent A to Eluent B. Injection volume of 5 µL at 1 mL/min flow rate with detection via UV Vis at 220 nm.

RP-HPLC of ARV-110: Column: Waters XBridge Column C8 (4.6 × 50 mm, 3.5 µm), oven: 37 °C; mobile phase: Eluent A = 0.1% formic acid in deionized water, Eluent B = 0.1% formic acid in acetonitrile, linear gradient of 90:10 Eluent A: Eluent B. Injection volume of 5 µL at 1.7 mL/min flow rate and UV Vis detection at 254 nm.

#### 2.2.8. Microtransfer

The raw material/spray-dried dispersion was suspended in SGF at pH 2.0 (0.1 mg/mL) using an ultrasonic bath. The stock solution was added to a heated vertical Franz diffusion cell (37 °C). Next, 3 mL of the solution was transferred into a different vertical Franz diffusion cell filled with FaSSIF DB at pH 6.5 within 30 min. For the transfer, a tube (0.89 mm) and 0.2 µm PTFE filters were used. After taking samples at 5, 10, 15, 30, 60 and 120 min, the diffusion cell was refilled with 200 µL of warmed FaSSIF DB at pH 6.5. The samples were analyzed using RP-HPLC.

#### 2.2.9. Activity Assay

Western Blot activity assays were performed, as previously described in the literature [18]. HEK293 cells endogenously expressing human FKBP51 were treated with different concentrations of the PROTAC SelDeg51, either straight from the original synthesis or the formulation mixture. After 24 h, cells were lyzed, and aliquots from the lysates were separated on an SDS gel and analyzed by Western blotting using an anti-FKBP51 antibody to determine the remaining FKBP51 protein levels in the cell lysates.

#### 2.2.10. Fluorescence Polarization Assay for VCB binding

PROTAC binding to the von Hippel–Lindau–ElonginC–ElonginB complex (VCB) was investigated using a competitive fluorescence polarization assay. Therefore, a stock solution of the PROTAC in assay buffer (20 mM of HEPES at pH 8.0), 150 mM NaCl and 0.015% Triton X-100 and 5% (*v*/*v*) PEG3350 supplemented with 6 μM of FKBP51^FK1^ was placed in a black 384 well assay plate (Greiner, Kremsmuenster, Austria). A serial dilution series of the PROTAC was performed in assay buffer supplemented with 1 µM of FKBP51^FK1^ and incubated with 6 nM of VCB complex and 1 nM of VHL-FP tracer [18]. After 30 min incubation at room temperature, the fluorescence polarization was measured with a TecanSpark (Ex.: 535 nm, Em.: 595 nm). The mean values for 3 replicates were fitted using a competitive binding model, as described by Kozany et al. [21].

#### 2.2.11. Stability Studies

For the stability test, the ARV-110 samples were stored in closed vials at 5 °C. After four weeks (t = 1 M), the samples were examined in XRPD and dissolution.

The SelDeg51 samples were stored in closed vials at 5 °C and analyzed after four weeks (t = 1 M) in XRPD and dissolution.

## 3. Results and Discussion

### 3.1. Spray Drying of Crystalline PROTAC ARV-110 with PVA

**Figure 1:**
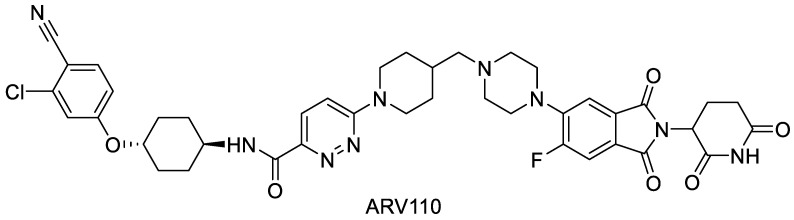
Molecular structure of ARV-110.

ARV-110 has a molecular mass of 812.3 Da and a cLogP of 4.26 and addresses the cereblon E3 ligase (Figure 1). It is currently in clinical trials for the treatment of metastatic castration-resistant prostate cancer. These patients usually have scarce options for treatment due to resistance or insensitivity to anti-androgenic therapies. As a well-tolerated dose, 420 mg of ARV-110 was evaluated, and the signs of antitumor activity in early trial outcomes showed its potential [22,23].

This compound was chosen as the model PROTAC in this study to evaluate the solubility enhancement of PROTACs by spray drying with Poly(vinyl alcohol), as it is an orally bioavailable candidate already in clinical testing. However, it displays very low aqueous solubility. Thus, solubility enhancement can be well investigated with this compound. Ultimately, a higher solubility could enhance its bioavailability, resulting in a reduction in the applied dose in the final drug product and decreasing the pill burden.

**Figure 2:**
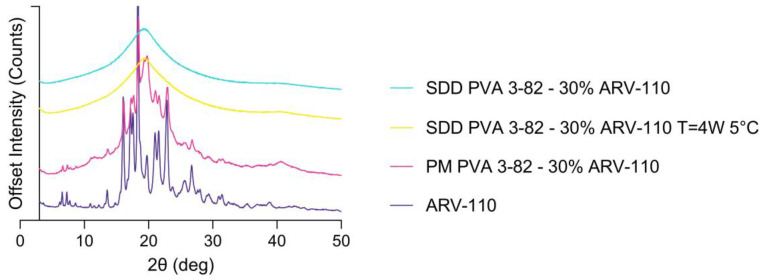
X-ray powder diffractograms of the PROTAC ARV-110 and the physical mixture (PM) of ARV-110 with PVA containing 30% drug load as well as the spray-dried dispersion (SDD) of ARV-110 with PVA containing 30% drug load at t = 0 and after storage for 4 weeks at 5 °C (t = 4 W 5 °C).

**Figure 3:**
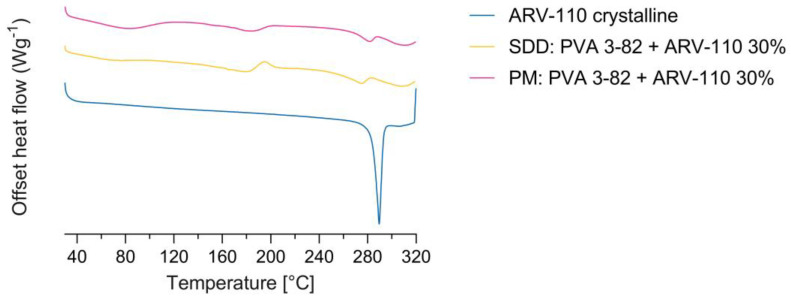
Differential scanning calorimetry of the PROTAC ARV-100, the physical mixture (PM) and the spray-dried dispersion (SDD) containing a 30% drug loading of ARV-110 in the PVA matrix.

Spray drying of the crystalline ARV-110 compound with Poly(vinyl alcohol) using a three-fluid nozzle approach [16] led to the successful amorphous embedding of the API with a 30% drug load in the polymer matrix, as shown in Figure 2 and Figure 3. The crude ARV-110, as well as the physical mixture containing 30% ARV-110 and 70% PVA, show intense peaks, indicating crystallinity at 16.08, 18.35 and 22.98 2θ deg in the diffractogram (Figure 2). In the spray-dried sample containing 30% ARV-110, these significant peaks are not detectable. This indicates successful amorphization of the crystalline ARV-110 through the spray drying process. Furthermore, the stability of the spray-dried dispersion was illustrated as the SDD sample still lacked crystalline peaks, especially at 16.08, 18.35 and 22.98 2θ deg, after 4 weeks of storage at 5 °C in the XRD diffractogram. Additionally, the amorphization of ARV-110 using spray drying can be determined by looking at the DSC results (Figure 3). The crystalline substance is characterized by a prominent solitary melting peak at T_m_ = 290 °C. For the physical mixture containing 30% *w*/*w* ARV-110 in the PVA matrix, a melting temperature with a slight shift to 285 °C can be determined. Additionally, in accordance with the literature data, the T_m_ of the semi-crystalline PVA 3-84 can be detected at 170 °C, as well as the glass transition temperature at 60 °C [24]. For the spray-dried sample, the thermogram changes compared to the crystalline substance and physical mixture, displaying a prominent exothermic peak at 200 °C. This peak could indicate ARV-110 underwent a change from an amorphous to a crystalline state during sample heating. A similar thermogram was presented by Lee et al., with amorphous Empagliflozin as a model compound manufactured by an ultrasonic nebulizer method [25]. Additionally, potential in situ crystallization and heat-induced sample alteration events of previously amorphous structures during heating in DSC measurements are described by Dedroog et al. [26]. The absence of two distinct glass transition temperatures excludes an amorphous phase-separated ASD [27]. These findings indicate that the DSC measurements of ASDs containing larger and flexible structures, such as PROTACs, pose challenges in distinct characterization.

**Figure 4:**
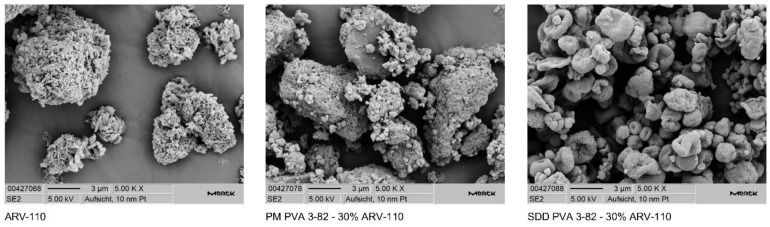
Scanning electron microscopy images of the PROTAC ARV-110 and the physical mixture (PM) of ARV-110 with PVA containing 30% drug load as well as the spray-dried dispersion (SDD) of ARV-110 with PVA containing 30% drug load at 5000× magnification.

Using SEM, a change in particle morphology indicates the absence of two individual substances in the spray-dried dispersion, as seen in the physical mixture (Figure 4).

**Figure 5:**
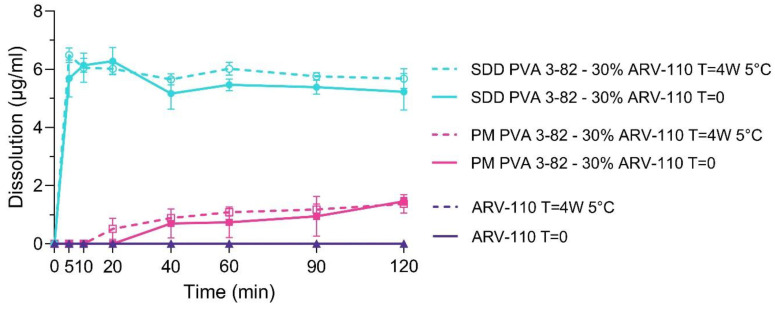
Dissolution profiles of the PROTAC ARV-110, the physical mixture (PM) of ARV-110 with PVA containing 30% drug, and the spray-dried dispersion (SDD) of ARV-110 with PVA containing 30% drug, as well as the respective materials, stored at 5 °C for 4 weeks in phosphate buffer with a pH of 6.8. Arithmetic means of n = 3 ± SD.

Dissolution of the neat ARV-110 API, the physical mixture (PM) of PVA and 30% ARV-110 and the spray-dried dispersion (SDD) of PVA with a 30% drug load of ARV-110 were investigated in a phosphate buffer with a pH of 6.8 (Figure 5).

Due to its low aqueous solubility and weakly basic behavior, neat ARV-110 displayed no detectable dissolution in the phosphate buffer with a pH of 6.8 over 120 min. By spray drying the PROTAC with Poly(vinyl alcohol) 3-82, solubility in the phosphate buffer was considerably enhanced for ARV-110. A fast onset within the first 5 min of measurement, as well as a steady state of dissolution over 120 min, showing a pronounced parachute behavior of the polymer, was demonstrated. Further, it was seen that the sole presence of PVA in the physical mixture slightly enhanced the solubility of the PROTAC in the phosphate buffer. Thereby, the physical mixture reaches a c_max_ of 1.6 µg/mL, and the SDD is 6.2 µg/mL. The stability of the SDD was highlighted as the dissolution profile of the SDD displayed comparably strong solubility enhancement effects of the spray drying with PVA after 4 weeks of storage at 5 °C. Again, for the crystalline ARV-110, no free drug could be detected within 120 min of dissolution in a phosphate buffer with a pH of 6.8.

**Figure 6:**
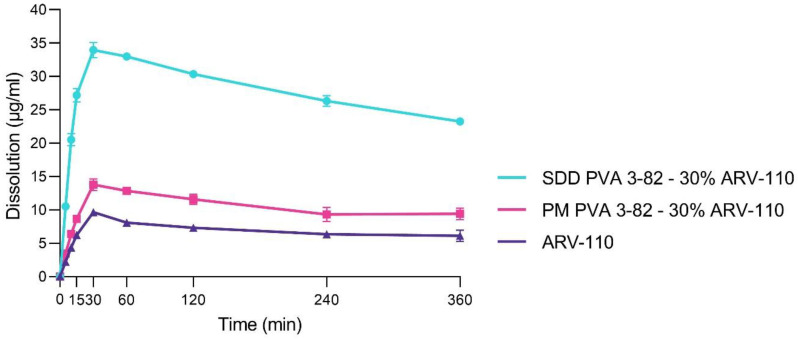
Dissolution profiles of the PROTAC ARV-110 and the physical mixture (PM) of ARV-110 with PVA containing 30% drug as well as the spray-dried dispersion (SDD) of ARV-110 with PVA containing 30% drug in fasted-state simulated intestinal fluid with a pH of 6.5 after transfer from simulated gastric fluid with a pH of 2.0. Arithmetic means of n = 2 ± SD.

To reflect the gastrointestinal environment more accurately, the dissolution profile of neat ARV-110, the physical mixture and the SDD was investigated in biorelevant media, following a transfer from simulated gastric fluid (SGF, pH 2.0) to fasted-state simulated intestinal fluid (FaSSIF, pH 6.5) to better predict potential in vivo behavior (Figure 6). In accordance with the results in the phosphate buffer, the solubility of the neat crystalline ARV-110 compound, which displayed low dissolution after 360 min in FaSSIF after transfer from the SGF, was strongly enhanced by spray drying with Poly(vinyl alcohol). A profound spring within the first 30 min, followed by a slight steady decrease in dissolution over 360 min, was demonstrated for the SDD. Further, the slight enhancement of solubility for ARV-110 by the presence of PVA in the physical mixture was confirmed again. As the maximum concentration (c_max_) of free ARV-110, 9.7 µg/mL of ARV-110 was found in the crystalline sample. For the SDD, a 3.5 times higher concentration of ARV-110 was detected, accounting for 34.0 µg/mL at c_max_. The sole physical mixture of PVA with ARV-110 was able to enhance dissolution 1.4 times, yielding 13.8 µg/mL of free ARV-110 at c_max_. Reflecting the weakly basic molecular structure, the c_max_ can be found at 30 min of the experiment, where a pH of 2 is present. Still, the re-crystallization of ARV-110 is inhibited by the presence of PVA in the SDD after the pH shift, even after 6 h, and the concentration of free ARV-110 in the SDD sample accounts for approximately 25 µg/mL, whereas for the crude product, only 5 µg/mL of free drug can be detected.

These findings extend the applicability of ASD formulations with PROTACs. One of the first reports on using ASDs with PROTACs was published by Poestges et al. with vacuum compression molding (VCM). They investigated EL 100-55 and HPMC-AS as polymeric matrices in which ARCC-4, an androgen receptor-targeting PROTAC, was amorphously embedded using a melting approach. Solubility enhancement and precipitation inhibition were determined for 10% and 20% drug loading (DL) in dissolution experiments in a phosphate buffer with a pH of 6.8 [28]. In a second report, Hofmann at al., show the applicability of spray drying PROTACs and polymers to generate ASD using the PROTAC MS4075 with Soluplus and E PO in a 10% DL [17].

### 3.2. Spray Drying of Amorphous PROTAC SelDeg51 with PVA

**Figure 7:**
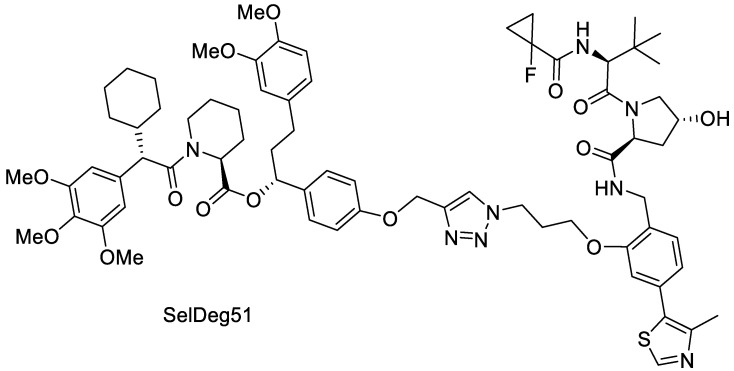
Molecular structure of SelDeg51.

Solubility enhancement by the production of amorphous solid dispersions is often based on the stabilization of the energetically higher amorphous state of a crystalline API in a polymer matrix. However, in contrast to the crystalline ARV-110, many of the currently developed PROTACs already display an amorphous solid state. Thus, another aspect of this study was to investigate if an initially amorphous PROTAC can be stabilized and its solubility enhanced when spray dried with Poly(vinyl alcohol). Herewith, we follow up on the initial findings by Hofmann et al. and Postges et al., who both investigated ASDs with initially amorphous PROTACs. Both provided insights that embedding an initially amorphous PROTAC into a polymeric matrix through an enabling technology (MeltPrep as well as spray drying) enhances the solubility significantly compared to the amorphous crude product. Therefore, SelDeg51 was used as a model compound due to its initial amorphous state and low aqueous solubility (Figure 7).

SelDeg51 utilizes the E3 ligase VHL and targets the FK506-binding protein 51 (FKBP51). FKBP51 is a key regulator of the human stress response and a potential target for depression, chronic pain and obesity [29]. Recently, it was shown that the FKBP51 PROTAC SelDeg51 can block the inhibitory effect of FKBP51 on the glucocorticoid receptor [18], which is not possible for occupancy-based ligands [30].

**Figure 8:**
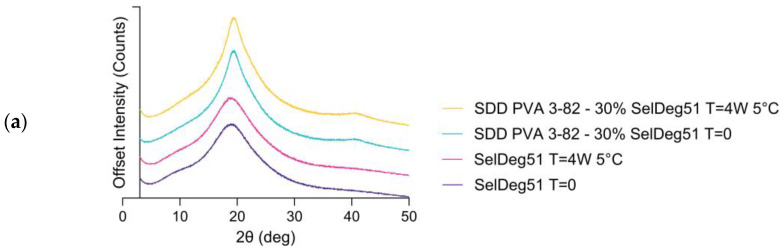

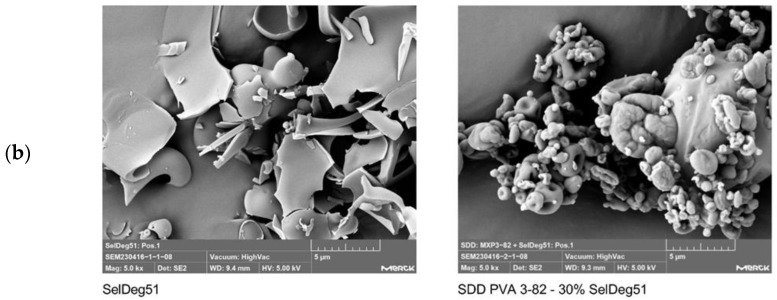
(**a**) X-ray powder diffractograms of the PROTAC SelDeg51 and the spray-dried dispersion (SDD) of SelDeg51 with PVA containing 30% drug load as well as the respective materials stored at 5 °C for 4 weeks and (**b**) scanning electron microscopy images of SelDeg51 and the spray-dried dispersion (SDD) of SelDeg51 with PVA containing 30% drug load at 5000× magnification.

SelDeg51 was successfully embedded in the polymer matrix by spray drying with Poly(vinyl alcohol) with a 30% drug load, again using the three-fluid nozzle approach, as shown by X-ray powder diffraction and scanning electron microscopy (Figure 8). The X-ray powder diffractograms demonstrate that the API was amorphous before and after spray drying, indicated by the amorphous halo. This amorphous state did not change within the storage time of 4 weeks at 5 °C (Figure 8a). The scanning electron microscopy images (Figure 8b) demonstrate the change in particle morphology before and after spray drying.

**Figure 9:**
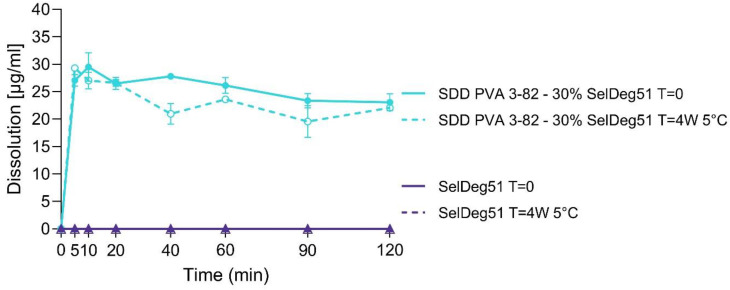
Dissolution profiles of the PROTAC SelDeg51 and the spray-dried dispersion (SDD) of SelDeg51 with PVA containing 30% drug as well as the respective materials stored at 5 °C for 4 weeks in phosphate buffer with a pH of 6.8. Arithmetic means of n = 3 ± SD.

The dissolution of SelDeg51 and the spray-dried dispersion (SDD) of PVA with a 30% drug load of SelDeg51 was investigated in a phosphate buffer with a pH of 6.8 (Figure 9).

Due to its low aqueous solubility in the respective pH, neat SelDeg51 displayed no detectable dissolution in the phosphate buffer over 120 min. By spray drying with Poly(vinyl alcohol) 3-82, solubility in the phosphate buffer was strongly enhanced for SelDeg51, which is in accordance with the results for the crystalline ARV-110. A fast onset within the first 5 min, as well as a steady decrease in dissolution over 120 min, showing a pronounced parachute behavior of the polymer, was demonstrated for the SDD of SelDeg51. A similar dissolution profile indicating an enhanced release was seen for the SDD after storage at 5 °C for 4 weeks, thus demonstrating the stability of the SDD. Again, for the crude API, SelDeg51, dissolution of the sample after 4 weeks at 5 °C did not result in any detectable amounts of free API for 120 min of dissolution.

Thus, it was highlighted that despite the initially already amorphous state of the PROTAC SelDeg51, the solubility was considerably enhanced by stabilizing the PROTAC in a polymer matrix by spray drying with Poly(vinyl alcohol). Again, these findings complement the previously mentioned successes in PROTACs–ASD design by Poestges and Hofmann et al. [17,28]. As PROTACs represent molecules of approximately 1000 Da with potential flexibility, especially due to their flexible linker sections, formulators will be faced with structures that display an initial amorphous state but, following the bRo5, pose challenges in oral formulation. As can be seen from these three research paper results, enabling formulations can be applied to these amorphous structures, enhancing solubility significantly.

**Figure 10:**
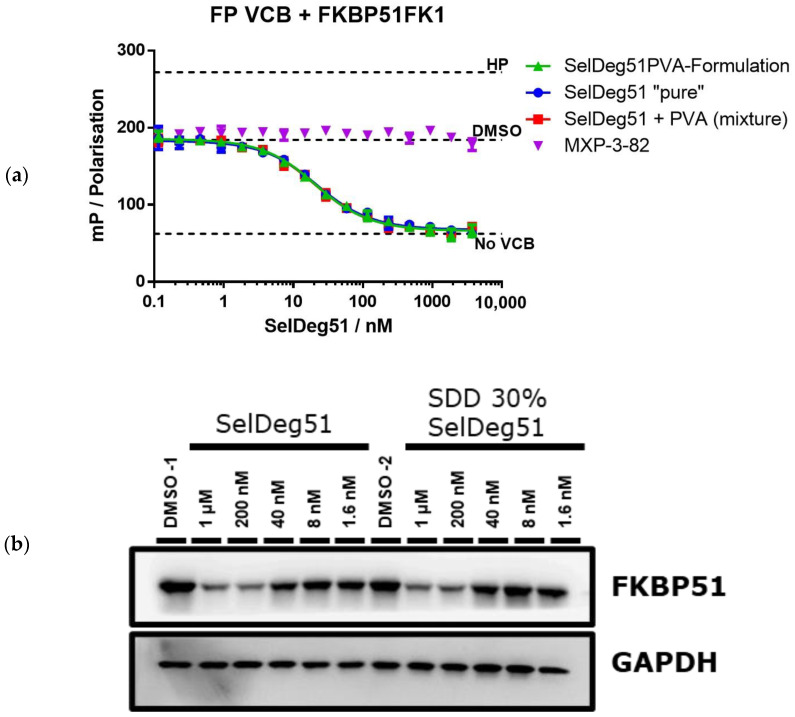
Performance characterization of SelDeg51 after spray drying. (**b**) Western blot analysis of SelDeg51-mediated FKBP51 degradation by neat SelDeg51 as well as the SDD with 30% drug load after 24 h of treatment in HEK293T cells. GAPDGH was determined as loading control. (**a**) Binding to the E3 ligase VHL as determined by fluorescence polarization of neat SelDeg51, the SDD, a physical mixture with 30% drug load and neat PVA in presence of a saturating excess of FKBP51^FK1^HP: high protein control representing fully bound VHL tracer; DMSO with no additional substance treatment; No VCB: Assay mix without VHL/EloB/EloC, representing fully unbound tracer.

To test the functional integrity of the released PROTAC, a fluorescence polarization assay was used to analyze binding to the E3 ligase VHL (in a complex with EloB and EloC) of neat SelDeg51, the SDD, a physical mixture with a 30% drug load and neat PVA (Figure 10a). This essay highlighted that the spray drying process had no negative impact on the protein binding of SelDeg51. Thus, the protein binding and degradation activity of SelDeg51 were fully maintained throughout and after the spray drying process with PVA.

Further, the FKBP51 degradation capacity of neat SelDeg51 as well as the SDD with a 30% drug load after 24 h of treatment in HEK293T cells was assessed by Western blot. This demonstrated that the spray drying process did not negatively impact the protein degradation activity of SelDeg51, as no difference between both samples is visible (Figure 10b).

## 4. Conclusions

PROTACs hold the potential to address undruggable targets in oncological, immunological and neurodegenerative diseases. However, their heterobifunctionality often leads to unfavorable properties critical for oral bioavailability, such as solubility and permeability. In this study, the solubility enhancement of two poorly soluble PROTACs via spray drying with Poly(vinyl alcohol) was investigated. A three-fluid nozzle spray drying process with PVA, with a hydrolysis degree of 82% and viscosity of 3 mPa × s (at 20 °C), was applied to manufacture amorphous solid dispersions with a 30% drug load of the initially crystalline PROTAC ARV-110 and the initially amorphous PROTAC SelDeg51. For both poorly soluble PROTACs, a significant dissolution enhancement with a fast onset as well as a prolongation and long-term stabilization of the solubility was achieved by spray drying with Poly(vinyl alcohol), and the stability of the spray-dried dispersion for at least 4 weeks was highlighted. Additionally, the functional assays of SelDeg51 indicate that neither the presence of PVA nor the spray drying process itself influences the functional integrity and degradation potential. Thus, although further PROTACs need to be analyzed, this study indicates that solubility enhancement by spray drying with Poly(vinyl alcohol) could be a promising strategy to raise the oral bioavailability of poorly soluble PROTACs.

## Data Availability

The original contributions presented in the study are included in the article, further inquiries can be directed to the corresponding author.
